# Levels of Spending and Resource Allocation to HIV Programs and Services in Latin America and the Caribbean

**DOI:** 10.1371/journal.pone.0022373

**Published:** 2011-07-22

**Authors:** Daniel Arán-Matero, Peter Amico, Christian Arán-Fernandez, Benjamin Gobet, José Antonio Izazola-Licea, Carlos Avila-Figueroa

**Affiliations:** 1 Promotion of Economic Development (PROMODE), Joao Pessoa, Brazil; 2 The Heller School for Social Policy and Management, Brandeis University, Boston, Massachusetts, United States of America; 3 Joint United Nations Programme on AIDS (UNAIDS), Geneva, Switzerland; 4 National AIDS Control Programme, Ministry of Health, Mexico City, Mexico; Kenya Medical Research Institute - Wellcome Trust Research Programme, Kenya

## Abstract

**Background:**

An estimated 1.86 million people are living with HIV in Latin America and the Caribbean (LAC). The region is comprised of mainly middle-income countries with steady economic growth while simultaneously there are enormous social inequalities and several concentrated AIDS epidemics. This paper describes HIV spending patterns in LAC countries including analysis of the levels and patterns of domestic HIV spending from both public and international sources.

**Methods and Findings:**

We conducted an extensive analysis of the most recently available data from LAC countries using the National AIDS Spending Assessment tool. The LAC countries spent a total of US$ 1.59 billion on HIV programs and services during the latest reported year. Countries providing detailed information on spending showed that high percentages are allocated to treatment and care (75.1%) and prevention (15.0%). Domestic sources accounted for 93.6 percent of overall spending and 79 percent of domestic funds were directed to treatment and care. International funds represented 5.4 percent of total HIV funding in the region, but they supplied the majority of the effort to reach most-at-risk-populations (MARPs). However, prevalence rates among men who have sex with men (MSM) still reached over 25 percent in some countries.

**Conclusions:**

Although countries in the region have increasingly sustained their response from domestic sources, still there are future challenges: 1) The growing number of new HIV infections and more people-living-with-HIV (PLWH) eligible to receive antiretroviral treatment (ART); 2) Increasing ART coverage along with high prices of antiretroviral drugs; and 3) The funding for prevention activities among MARPs rely almost exclusively on external donors. These threats call for strengthened actions by civil society and governments to protect and advance gains against HIV in LAC.

## Introduction

Though Latin America is regarded as a ‘low prevalence’ region, the estimated 1.86 million people living with HIV will impose significant challenges in the coming years for the provision of health and social services [1], [2]. The 0.6 percent prevalence among HIV-infected adults in Latin America and the current stabilization of infections does not reflect the growing epidemic that has been unfolding over the last 30 years. Additionally, there has been a 22 percent increase in new infections between 2001 and 2009 which points to strong preventive programs in the region [7]. In 2008, Hotez et al. estimated that HIV accounts for 3.8 percent of the burden of disease in LAC [8].

The Caribbean is the second hardest hit region in the world with an infection rate of 1 percent and is a mix of generalized (>1% prevalence) and concentrated epidemics [9]. HIV is equally distributed between men and women, but higher prevalence is found among young women [9]. The regional infections seems to have stabilized with countries such as the Dominican Republic and Haiti experiencing declines in HIV prevalence [9]. Surveys in the region indicate high infection rates among sex workers ranging from 9 to 27 percent [9], [10]. Various studies of MSM infection rates range from 20 to 31 percent in Jamaica and Trinidad and Tobago respectively [9]. Though IDUs play a small role in the epidemic in the Caribbean, it is the main mode of transmission in Puerto Rico [9].

The Latin America and the Caribbean (LAC) regions are exiting the economic crisis faster than expected due to solid macroeconomic policy fundamentals, favorable external financing conditions and strong commodity revenues [11]. The projected growth for the region is 5.7 percent in 2010 and 4 percent in 2011 [11]. As other emerging economies, the gap between rich and poor is rising, situating LAC as the home to the world's most unequal societies; Brazil, Argentina and Chile all have GINI coefficients over 50 [12]. Social investment in Latin America during the last 30 years have been directed to transferring resources to the poor, creating temporary jobs and investing in education and health in order to soften the effects of the adjustment policies of the 1980s.

LAC has transitioned to more rapidly urbanized megacities along with the rise of slums and extreme urban poverty within these cities [13], [14]. In fact, Mexico City, Sao Paulo, Buenos Aires and Rio de Janeiro are all larger than 10 million people. More than five million inhabitants populate five other cities: Bogota, Lima, Santiago de Chile, Bello Horizonte and Guadalajara [13]. While, this allows more people to be in proximity of health services, urbanization is also associated with the adoption of unhealthy lifestyles—poor diet, obesity, lack of physical activity, alcohol and drug use, risky sexual behavior, and increasing violence and trauma [14]. Chronic non-communicable diseases are also contributing substantially to overall mortality and disease burden in the region [15].

National health expenditures in LAC represented approximately 6.8 percent of GDP or an annual per capita expenditure of US$ 500 [14]. Domestic funding directed to health systems and HIV in Latin America has been growing; in fact, there are 27,000 facilities providing HIV testing services with more than 5 million people tested in 2009. Anti-retroviral (ARV) coverage is widespread with a reported 478,000 people receiving ART in LAC. On average, ARV coverage has increased from 10 percent in 2004 to 50 percent in 2009 [7]. Though almost one million people need Anti-retroviral treatment (ART); LAC pays ARV prices that are above the global average [5].

The relatively low prevalence in Latin America is also diverting the attention from the high prevalence concentrated epidemics affecting specific groups [16]. The number of infections among men is much higher than among women in the region; for example, in Peru, the number of new male cases was three times the number of female cases in 2008 [16]. Men who have sex with men (MSM) have a one in three chance of being infected with HIV in Latin America and account for the largest share of HIV infections in the region [16]. Additionally, an estimated 29 percent of IDUs in Latin America are infected with HIV, though these infections are concentrated in the Southern Cone of South America and along the Mexico US border [16], [17]. The majority of transmission in LAC is through unprotected sex including MSM and both male, female and transgender sex workers, and there is growing concern over the spread among injecting drug users (IDUs) [1]. Cultural issues that have stigma, create dangerous opportunities for HIV to continue unabated. In LAC countries there is still a significant stigma associated with the disease which has hampered efforts to achieve universal access to prevention, treatment and care [18].

Monitoring the flow of resources for the HIV response provides valuable strategic information that can improve operations and planning, and mobilize greater resources. In regions where the funding gap is increasing, the mapping of HIV expenditures provides crucial guidance for the reallocation of resources and supports evidence-based decision making. Funding information also provides an indication of a country's commitment to tackling HIV, measured by domestic spending, and international support for the HIV response, as measured through donor contributions.

Using the most recent available data, this paper describes HIV spending in LAC countries. This includes analysis of the levels and patterns of domestic HIV spending from public and international sources and spending on most-at-risk populations, taking into account country-income levels.

## Methods

We conducted a descriptive analysis of HIV expenditures from 23 LAC countries using the most recently reported year—either 2008 or 2009. These countries included: Argentina, Belize, Bolivia, Brazil, Chile, Colombia, Costa Rica, El Salvador, Honduras, Mexico, Nicaragua, Panama, Paraguay, Peru, and Venezuela. The Caribbean countries include Antigua and Barbuda, the Bahamas, the Dominican Republic, El Salvador, Grenada, Saint Kitts and Nevis, Saint Vincent and the Grenadines, and Trinidad and Tobago. The available LAC data consists of 23 countries—15 from Central and South America, 8 from the Caribbean.

We also conducted a trend analysis for those countries with more than five reported points in time of domestic AIDS spending in Latin America. These countries have conducted systematic and standard resource tracking of AIDS spending since the mid 1990s [6].

All expenditures on HIV were generated from reporting on the United Nations General Assembly Special Session (UNGASS) indicator number 1 and the National AIDS Spending Assessment (NASA) tool which was developed by UNAIDS to measure all the resources included in a country's national HIV response and was developed using the national health accounts framework [3], [4]. These expenditures were cross- tabulated by source of financing and stratified by income level. These reports are generated by national resource tracking teams and do not include out-of-pocket or other types of private spending.

NASA applies standard accounting methods to reconstruct all transactions in a given country, following the money from the funding sources to agents and providers, and eventually to beneficiary populations. HIV spending is structured into eight categories of spending: (1) prevention; (2) treatment and care; (3) orphans and vulnerable children; (4) program management and administration; (5) human resources; (6) social protection; (7) enabling environment; and (8) research [19], [20].

We also estimated the AIDS priority index, with the objective of measuring a country's ability to fund its own AIDS response and the ability to sustain a long-term response. The index is estimated by calculating each country's percentage of government revenue directed to the AIDS response divided by HIV prevalence [7]. A high value usually indicates a high level of priority. If a country is spending at least the average in relation to their resources and HIV prevalence, it is giving a relatively high priority to AIDS.

Countries were classified by income level; economies were divided according to their 2009 Gross National Income (GNI) per capita, calculated using the World Bank Atlas Methods [21] and grouped into four categories: low-income (US$ 975 or less); lower middle-income (US$ 976–$3,855); upper middle-income (US$ 3,856–$11,905); and high income (US$ 11,906 or more). There are 9 lower middle-income countries (Belize, Bolivia, Colombia, El Salvador, Guatemala, Honduras, Nicaragua, Paraguay, Peru), ten upper-middle income countries (Argentina, Brazil, Chile, Costa Rica, Dominican Republic, Grenada, Mexico, Panama, Saint Vincent and the Grenadines and Venezuela), and four high income countries (Antigua and Barbuda, the Bahamas, Trinidad and Tobago, and St. Kitts and Nevis) [21], [22]. Countries with no reports from 2008–9 were excluded.

## Results

The 23 LAC countries spent a total of US$ 1.59 billion on HIV programs and services during the last reported year. Of those 23 countries, 20 provided detailed information on their levels of spending within each of the eight different HIV spending categories. As Table 1 reports, treatment and care received the largest share of funds (75.1%), with the remaining resources divided between prevention (15.0%), program management and administration strengthening (4.0%), creating an enabling environment (2.0%), human resources (1.7%), research (.5%) and orphans and vulnerable children (.1%).

**Table 1:** Reported total, international and domestic spending on HIV spending categories in 20 LAC countries (latest year available).

HIV Spending Categories	Total Spending	Percent of Spending	Total Public Spending	Total Int'l Spending	Percent International
1. Prevention	$ 237,745,926	15.0%	$ 193,134,352	$ 39,943.029	17%
2. Care and Treatment	$ 1,190,983,632	75.1%	$ 1,171,664,929	$ 17,057,633	1%
3. Orphans and Vulnerable Children	$ 2,126,125	0.1%	$ 561,244	$1,535,738	72%
4. Program Management and Administration Strengthening	$ 63,742,356	4.0%	$ 43,686,487	$17,627,592	28%
5. Human resources	$ 27,330,314	1.7%	$ 19,662,261	$2,949,854	11%
6. Social Protection and Social Services excluding Orphans and Vulnerable Children	$ 22,939,222	1.4%	$ 22,607,171	$ 332,051	1%
7. Enabling Environment	$ 32,019,116	2.0%	$ 23,766,462	$7,715,162	24%
8. Research	$ 8,644,643	0.5%	$ 4,359,132	$3,935,298	46%
TOTAL	$ 1,585,531,333	-	$ 1,479,442,039	$ 91,096,357	-

Table 1, which lists the proportional allocation of international and public resources for HIV, shows that international resources are the dominant funding source for orphans and vulnerable children (72%) followed by research (46%) and program management (28%). Domestic resources are the primary funding source of treatment and care (99%) followed by social protection (99%) and prevention (83%). However, International funds are a relatively minor part of HIV spending in many LAC countries and overall account for 6% of the total funding (Table 2).

**Table 2:** Reported total and per capita per year spending and proportion of international funding in 20 LAC countries (latest year available).

Country	Year	Income Level	% International Funding	Total HIV Spending	HIV Spending per Capita	Epidemic State	PLWHIV	HIV Spending per PLWHIV
Grenada	2009	Upper middle	0%	$ 194,493	$ 2.62	L	403	$ 483
Venezuela	2009	Upper middle	0%	$ 78,800,637	$ 2.89	C	136,625	$ 577
Colombia	2009	Lower middle	1%	$ 108,791,907	$ 4.03	C	173,911	$ 626
Mexico	2009	Upper middle	1%	$ 218,421,242	$ 3.35	C	215,563	$ 1,013
Chile	2008	Upper middle	1%	$ 88,012,301	$ 7.34	C	31,811	$ 2,767
Brazil	2008	Upper middle	1%	$ 623,133,515	$ 3.82	C	391,257	$ 1,593
Argentina	2008	Upper middle	3%	$ 248,772,695	$ 10.82	C	122,074	$ 2,038
Costa Rica	2008	Upper middle	7%	$ 19,884,919	$ 7.28	C	9,953	$ 1,998
Bahamas	2009	High income	10%	$ 4,888,516	$ 16.30	G	6,477	$ 755
Saint Kitts and Nevis	2009	Upper middle	11%	$ 1,210,091	$ 29.67	C	314	$ 3,854
Panama	2008	Upper middle	13%	$ 13,627,719	$ 7.24	C	20,351	$ 670
Trinidad and Tobago	2009	High income	16%	$ 13,532,974	$ 15.69	C	13,962	$ 969
El Salvador	2008	Lower middle	20%	$ 39,227,433	$ 11.98	C	35,975	$ 1,090
Paraguay	2009	Lower middle	32%	$ 11,417,737	$ 3.31	C	22,118	$ 516
Antigua and Barbuda	2009	High income	34%	$ 390,760	$ 6.64	C	815	$ 479
Peru	2009	Lower middle	45%	$ 43,638,623	$ 2.88	C	80,281	$ 544
Nicaragua	2008	Lower middle	58%	$ 14,908,986	$ 5.95	C	7,866	$ 1,895
Honduras	2008	Lower middle	62%	$ 24,319,656	$ 6.44	C	28,803	$ 844
Dominican Republic	2008	Lower middle	65%	$ 23,415,929	$ 3.95	C	62,009	$ 378
Belize	2009	Upper middle	68%	$ 2,024,335	$ 9.68	C	3,957	$ 512
Dominica	2009	Upper middle	83%	$ 177,655	$ 4.22	C	350	$ 508
Bolivia	2009	Lower middle	87%	$ 7,418,172	$ 1.83	L	8,562	$ 866
Saint Vincent/Grenadines	2009	Upper middle	92%	$ 2,629,219	$ 42.22	C	427	$ 6,157
Total			6%	$ 1,588,839,511	$ 9.14		1,373,864	$ 1,156

Most countries funded their own HIV response; domestic sources accounted for 93.6 percent of overall spending. Domestic spending ranged from 100 percent in Grenada to 8.4 percent in Saint Vincent and the Grenadines. Notably, upper middle-income countries such as: Venezuela, Colombia, Mexico, Chile and Brazil funded more than 99 percent of their HIV response. However, seven countries rely on external support to fund over half of their response to HIV including: Nicaragua, Honduras, the Dominican Republic, Bolivia, Belize, Dominica and Saint Vincent and the Grenadines. External support made up 43 percent of the total spending in the eight lower middle- income countries, 23 percent in the 13 upper middle- income countries and 19 percent in the three high- income countries.

The average growth rate for the two latest available years of data was 12 percent. Of the countries observed, only four experienced declines in the total spending for HIV: Grenada, Mexico, Saint Kitts and Nevis and Trinidad and Tobago. All of these countries cut their domestic spending on HIV between 2008 and 2009. Mexico also experienced a drop in international funding during the last year reported while the other countries continued to receive additional international support. International funding grew at an average rate of 34 percent with only Mexico, Peru and Venezuela experiencing declines. Brazil and Argentina showed the largest expansion of funds in absolute terms, with resource levels increasing by US$ 47.9 million and US$ 39.3 million respectively between 2007 and 2008. For those countries whose funding increased, they ranged from 3 percent in El Salvador to 62 percent in Saint Vincent and the Grenadines.

Spending patterns varied across all countries, as reflected in Table 2, which shows total and per capita spending, and the proportion of international contributions. Brazil (US$ 623.1 million) is the largest spender in absolute terms, trailed by Argentina (US$ 248.7 million) and Mexico (US$ 218.4 million). Saint Vincent and the Grenadines is the biggest per capita spender at US$ 42.22 per capita. Annually, countries spent US$ 9.14 per capita on HIV and US$ 1,156 per person living with HIV (PLWH), however, this was mostly driven by the high spending in countries with low numbers of PLWH which have higher fixed costs.

Although treatment and care received 73 percent of total resources, Belize, Bolivia, and Honduras allocated a greater proportion of resources to prevention than treatment and care, allocating 27, 37 and 59 percent respectively. The highest proportion of spending on treatment and care occurred in Venezuela with 88 percent of the funds going to treatment and care. Several countries spent a very high percentage of their spending on program management and administration. The Bahamas, the Dominican Republic, Belize, and St. Kitts and Nevis spent 22.8, 39, 40 and 83 percent respectively. This was primarily driven by external support in Belize, Bolivia and the Dominican Republic. However, in the highest spender, St. Kitts and Nevis, domestic funding drove this large share.

**Figure 1:**
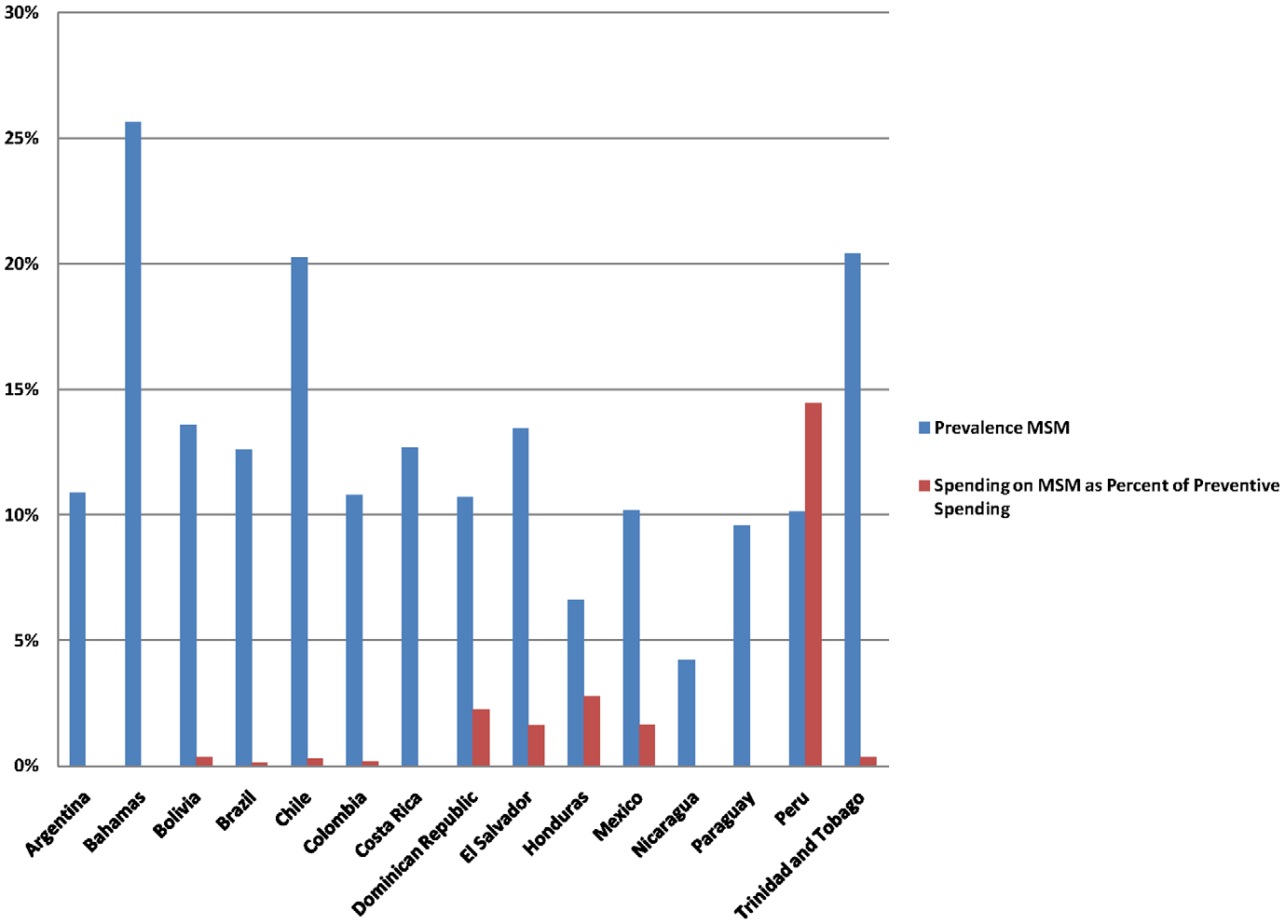
Prevalence among MSM and Spending on MSM as a Percent of Preventive Spending.

The most at risk populations (MARPs) in the HIV epidemic in LAC include sex workers and their clients, men who have sex with men (MSM) and injecting drug users (IDUs). Colombia, Mexico, Venezuela and Chile are the only countries who domestically fund outreach to MARPs and these funds are only 1, 3, 5 and 12 percent of overall prevention spending respectively. The remaining countries have either no funding for MARPs or are completely reliant on external donors. Peru has the highest percentage of prevention spending targeted at MARPs with 36 percent while 9 of the 20 countries with detailed reporting have no funding targeted at MARPs. As seen in Figure 1, the proportion of preventive spending allocated towards MSM is relatively low in most countries, especially in comparison to HIV prevalence rates among MSM.

Anti retroviral treatment (ART) is an important component of domestic HIV funding in LAC. Most of the funding to support ART comes from domestic sources; these represent 75 percent of total domestic funding for HIV. The only country that primarily relied on donor support for ART was Paraguay, who received 71 percent of their funding from external sources. However, the remaining countries funded 85 percent or more of their ART response with many countries funding 100 percent of the effort. The average LAC country spent 47 percent of their care and treatment budget on ART, ranging from 9 percent in the Bahamas to 93 percent in Chile.

**Figure 2:**
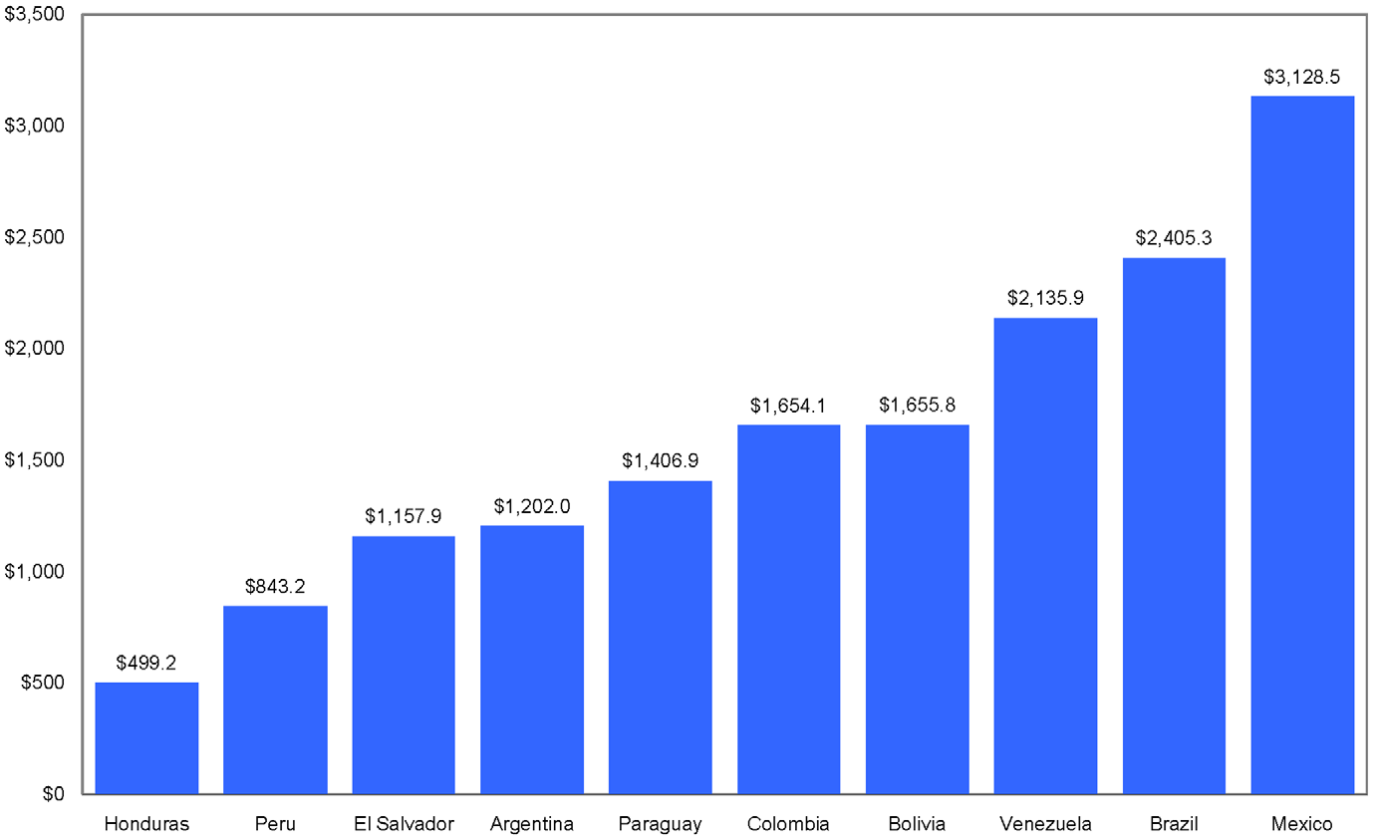
Treatment and Care Spending Per Person on ART in 9 LAC Countries in 2008.

Figure 2 shows 9 countries' total spending on care and treatment divided by the number of people on ART in the country. On average, ART was responsible for half of the care and treatment budget. Therefore, these do not represent the unit costs of ART, but rather the cost per person on treatment and care including drug and non-drugs costs. There is wide variation in the average cost of treatment ranging from US$ 843 in Peru to US$ 3,128 in Mexico.

**Figure 3:**
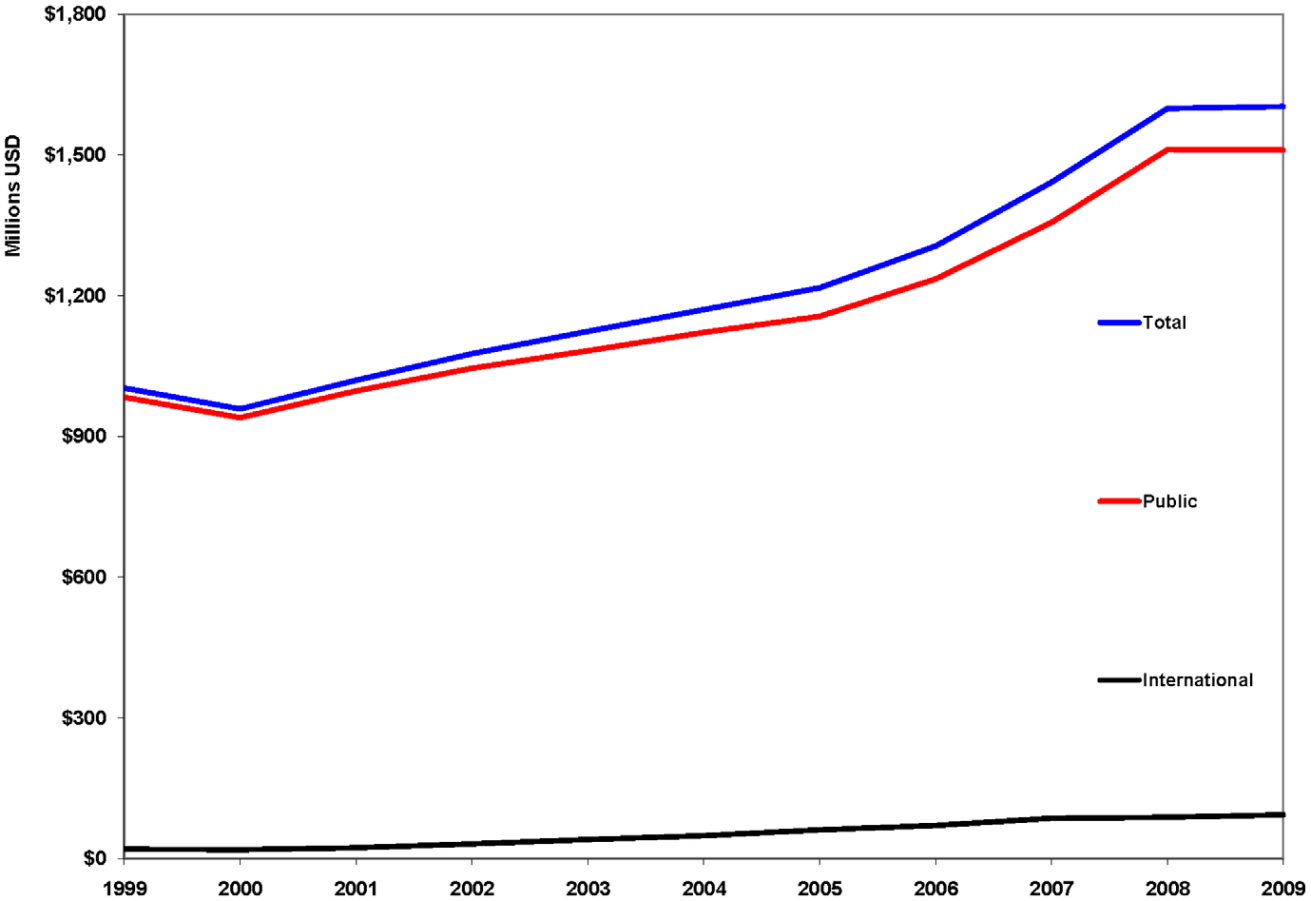
Public HIV Spending in 16 LAC Countries 1999–2009.

The overall trend in AIDS domestic spending (Figure 3) shows that, in 16 LAC countries, there was a nearly 60 percent increase in AIDS financing between 1999 and 2009, equivalent to an annual growth of 6%. In 2008, overall funding essentially remained flat, growing by less than 0.5 percent. Domestic funding accounts for the majority of funding in the region and international support is mostly targeted at low-income countries.

**Figure 4:**
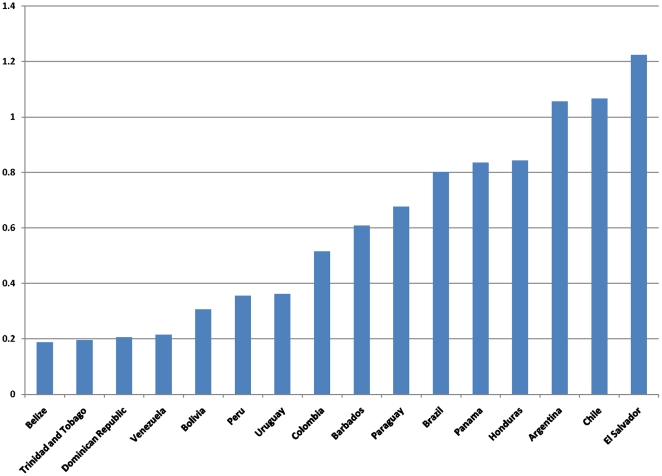
AIDS Priority Index by Country.

As illustrated in Figure 4, the domestic priority HIV index shows a country's ability to fund its own AIDS response. The median domestic priority-spending index of 0.68 as estimated for 19 countries in the region; ten countries are spending above the average in the region in relation to their resources and HIV prevalence, thus giving relatively high priority to AIDS.

Spending in eight countries, Argentina, El Salvador, Mexico, Brazil, Chile, Honduras, Panama and Nicaragua, exhibit a relatively high degree of priority given to HIV. On the contrary, Paraguay, Barbados, Colombia, Uruguay, Peru, Bolivia, Venezuela, the Dominican Republic, Belize, and Trinidad spend less than average in relation to their resources and HIV prevalence. These countries seem to have the potential to increase their domestic spending.

## Discussion

While this region accounts for 5% of the global number of people living with HIV [7], [23], the estimated US$ 1.59 billion spent in the 23 countries analyzed here represents 10 percent of the US$ 15.9 billion available for HIV in low- and middle-income countries in 2009 [7]. The US$ 1.59 billion spent in the region falls below the US$ 3.1 billion needed to achieve HIV universal access targets in Latin America and the Caribbean by 2010 [7], [24]. Also, the average LAC country spent 1.1 percent of their overall health budget on HIV related activities [25].

Resources for HIV have generally been on the rise in the region in recent years. According to internally collected NASA data, spending has grown at an annual rate of 6 percent in 16 countries reporting data for 10 years with few experiencing declining growth rates of HIV resources. The epidemic is stable or decreasing in all of the LAC countries which provides an opportunity to scale up HIV funding to meet funding needs [7]. Brazil and Argentina showed the largest increases in funding while Cuba and Saint Vincent and the Grenadines had the largest percentage growth. This is an encouraging trend, especially in the presence of a stabilizing or declining epidemic and steady regional economic growth.

Treatment represents 79% of public spending on AIDS in LAC and about half of this amount is invested in antiretroviral drugs. An additional burden comes from the fact that ART prices are above the global average in LAC [5]. Veronika Wirtz, et al. proposed that this is largely due to procurement methods and donor policies. As LAC has not been the focus of major price-reducing efforts in the same way as Sub-Saharan Africa, the prices may have increased due to the ability to pay [5]. This is a threat to scale-up efforts and to the future sustainability of HIV treatment [26]. The LAC countries could increase their market power by purchasing as one unit, creating a regional purchasing entity and incorporating generics [27].

The LAC region has not benefited from reductions in the prices of first-line regimens; the weighted median price of the six most widely used first-line regimens was US$ 137 per person per year in low-income countries. However, in LAC, the cost of first-line regimens is comparable to the US$ 202 paid by middle-income countries and to the US$ 1,378 paid for second line regimens. In the Americas Region, at least 10 percent of patients are being treated with a second-line regimen and an additional six percent are on salvage therapy. This is most likely due to the relatively longer duration and maturity of antiretroviral therapy programs in LAC's largest countries [28]. This may also help to explain the relatively higher costs of ART programs in LAC [29].

Since the largest source of HIV funding in LAC is domestic expenditure; the domestic priority HIV index shows a country's ability to fund its own AIDS response. More than half of the countries are spending above the average in the region in relation to their income and HIV prevalence, thus giving relatively high priority to AIDS. It is also possibly that this is a result of higher unit costs, especially for ARVs. The other half of countries would seem to have potential to increase their domestic spending sustainably. Colombia and Venezuela, two countries in South America with relatively high HIV prevalence, are spending at relatively low levels given their disease burden and ability to pay; thereby they could contribute more to the AIDS response from domestic resources.

Although domestic funding was the largest overall source of funding, due mainly to high domestic funding levels in Argentina, Brazil, Colombia and Mexico; over 40 percent of the countries receive more than one-third of their funding from international sources. In fact, seven countries receive more than half of their funding from non-domestic sources. External funding is particularly high for MARPs, where it accounts for almost all expenditures. It is worth noting that only a limited number of donor governments and philanthropic organizations invest in harm reduction approaches, raising the possibility that these approaches could be left even more underfunded if resources from international donors are reduced. The Global Fund is not a major player in the region as it has provided small grants to a few countries, most recently Mexico [30]. However, they have the potential to become an important resource in the region, especially if they support marginalized populations that are left behind by public programs.

Expenditures for prevention are low and represent only 15% of total spending in the region, especially under-funded are programs to prevent the expansion of concentrated epidemics and targeting MARPs—only 4 percent of total spending for prevention. In fact, this 4 percent of spending is almost fully from external donors rather than domestic funding sources. Although it is widely acknowledged that a significant portion of the transmission in LAC is through MSM, Sex workers and in some regions, IDUs; LAC countries are unwilling to fund prevention programs targeted at these MARPs. Some countries, such as Mexico have recently implemented harm reduction programs for high-risk populations [31].

Despite the fact that the HIV epidemic in LAC is driven by MARPs, especially MSM and sex workers, the majority of prevention spending is not targeted at these groups [32]. This funding mismatch points to a critical decision to reallocate scarce funding resources to where they can be most effective. In LAC countries, there is still a pervasive belief that HIV is a moral infection, thus governments are hesitant to fund targeted prevention efforts due to the stigma associated with these MARPs [18]. In order to continue making progress against the HIV epidemic more resources must be strategically focused on MARPs.

Failure to address the role of MSM and sex workers in HIV transmission could have serious health outcomes in the region. A study by Aldridge et al., found that cost-effectiveness of interventions among these groups ranged from US$ 55 to 5,928 per DALY averted in Peru [32]. Surveys in the region indicate infection rates ranging from 9 to 27 percent [9], [16]. Additionally, along the US-Mexico border and the southern cone of South America there is a high HIV infection rate among IDUs, 29 percent, which is of growing concern [16]. The data produced for this analysis clearly show that many LAC countries are not allocating their HIV resources in ways that are likely to achieve the greatest possible impact, particularly with respect to injecting drug users and other MARPs. Despite the significant political hurdles in addressing MARPs, strategic and evidence-based allocation of resources is even more critical during the global economic downturn, when it is probable that both national budgets and international contributions will remain flat or decrease.

The economic crisis has created widespread concerns that funding shortages will have an impact on prevention programs that work with stigmatized and marginalized population groups. LAC has emerged from the crisis relatively unscathed and has returned to positive growth [11]. It is expected that condom distribution and programs for IDUs could be seriously affected due to cost constraints and the immediacy of ART taking priority. However, the consequences of ignoring preventive programs will have a long-term impact in the LAC countries. The region also may expect negative impacts on national ART efforts and a decrease in financial resources for ART over the next year. A substantial contraction in the regional economy, plus devaluations of local currencies, could force some governments to cut overall public spending affecting the resources of national health insurance funds, which in some countries cover AIDS treatment costs.

The LAC countries have a long history of social security; coverage generally reflects the proportion of those workers who are employed in the formal sector—30–60 percent with the exception of some Caribbean islands where the formal economy is bigger [33]. It is estimated that approximately 140 million people do not have health insurance in LAC [34]. The uninsured population is at risk of catastrophic health expenditures, high out-of-pocket expenditures on health and the under-utilization of necessary health services [35], [36]. On average, households pay about one quarter of the total financial burden of HIV/AIDS expenses in LAC [37]. Households in Peru, Honduras, Paraguay, Uruguay and Belize take a share or more than one third of HIV/AIDS expenses; Peru is by far the largest with a share of nearly 80 percent [37]. Colombia, Guatemala, Mexico, Guyana, Panama and Venezuela have low shares of out-of-pocket spending on HIV—less than 15 percent [37]. The sustainability of the domestic response to HIV is in question if a high number of people are expected to pay out-of-pocket expenses for treatment.

This study is based on secondary analysis and has some limitations, as country reports may be incomplete and subject to variable levels of measurement error. Expenditures are estimated from different sources of information and some countries lack comprehensive and regular expenditure records and accounting information systems. The variation of NASA measurements across countries may limit the accuracy of the data. However, monitoring and evaluation officers in the region have improved the substantive basis for estimates of financial resources, while also working closely with countries to generate reliable data through the NASA resource tracking methodology [38]. Our analysis is limited to external and government sources of funding, neither of which include out-of-pocket or other private forms of household and business spending. Additionally, this analysis was only able to analyze total expenditures for 23 of the 26 LAC countries and detailed expenditures for 20 of the 26 LAC countries, thus our figures are slightly conservative for the region. This is not seen as a major limitation because the largest economies were all included.

The top spender in the LAC region, Brazil is also an upper-middle income country and is one of the four emerging key emerging economies referred to as BRICs (Brazil, Russia, India and China) with solid economic growth and an increasing share of the global gross domestic product. Brazil was the first middle-income country worldwide to guarantee universal access at no cost at the point of delivery of ARV treatment [2]. Brazil has also developed a generic drug industry and has been negotiating for greater use of the flexibilities regarding public health within the Trade-Related Intellectual Property Rights Agreement [39]. Brazil also has been engaged with the Global Fund; however, its disbursements of US$ 36 million are specifically for tuberculosis and malaria [40], [41], [42]. If Brazil ceases to be a recipient of international aid and shifts their support to less developed countries, then Brazil can play a major role and contribute to the global health policy agenda in LAC.

There are several threats to future sustainability in the current AIDS response: 1) an increasing number of new HIV infections along with more people-living-with-HIV reaching eligibility to receive ART, 2) an increasing coverage of ART, responsible for a large share of spending in LAC, and high prices paid for first and second line ARVs, 3) low income countries in the region receive more than one-third of their funding from international sources, and 4) prevention activities for MARPs are not only domestically under-funded, but also heavily reliant on external support.

Priority attention for the national responses in LAC should be given to ensuring treatment and care is included in social insurance schemes as well as an intensified focus on prevention. Also, strategic campaigns should be launched with the goal of reducing stigma and discrimination and improving social marketing to induce demand of preventive services. Governments should also develop strategies to move toward financial sustainability of AIDS programs while simultaneously increasing transparency and accountability. These objectives can only succeed with an engaged civil society and government partnerships to protect and advance gains against HIV in LAC.
